# Novel Conformation-Dependent Tau Antibodies Are Modulated by Adjacent Phosphorylation Sites

**DOI:** 10.3390/ijms241813676

**Published:** 2023-09-05

**Authors:** Giavanna Paterno, Jose Torrellas, Brach M. Bell, Kimberly-Marie M. Gorion, Stephan S. Quintin, Gabriela P. Hery, Stefan Prokop, Benoit I. Giasson

**Affiliations:** 1 Department of Neuroscience, College of Medicine, University of Florida, Gainesville, FL 32610, USA; giavanna@ufl.edu (G.P.); jtorrellas@ufl.edu (J.T.); brachbell@ufl.edu (B.M.B.); kimgorion@ufl.edu (K.-M.M.G.); squintin@ufl.edu (S.S.Q.); 2 Center for Translational Research in Neurodegenerative Disease, College of Medicine, University of Florida, Gainesville, FL 32610, USA; ghery01@ufl.edu (G.P.H.); sprokop@ufl.edu (S.P.); 3 Department of Pathology, College of Medicine, University of Florida, Gainesville, FL 32610, USA; 4 McKnight Brain Institute, College of Medicine, University of Florida, Gainesville, FL 32610, USA

**Keywords:** tau, antibodies, phosphorylation, conformation, pathological, neuropathology

## Abstract

Tau proteins within the adult central nervous system (CNS) are found to be abnormally aggregated into heterogeneous filaments in neurodegenerative diseases, termed tauopathies. These tau inclusions are pathological hallmarks of Alzheimer’s disease (AD), Pick’s disease (PiD), corticobasal degeneration (CBD), and progressive supranuclear palsy (PSP). The neuropathological hallmarks of these diseases burden several cell types within the CNS, and have also been shown to be abundantly phosphorylated. The mechanism(s) by which tau aggregates in the CNS is not fully known, but it is hypothesized that hyperphosphorylated tau may precede and further promote filament formation, leading to the production of these pathological inclusions. In the studies herein, we generated and thoroughly characterized two novel conformation-dependent tau monoclonal antibodies that bind to residues Pro218-Glu222, but are sensitive to denaturing conditions and highly modulated by adjacent downstream phosphorylation sites. These epitopes are present in the neuropathological hallmarks of several tauopathies, including AD, PiD, CBD, and PSP. These novel antibodies will further enable investigation of tau-dependent pathological inclusion formation and enhance our understanding of the phosphorylation signatures within tauopathies with the possibility of new biomarker developments.

## 1. Introduction

Tau is a microtubule-associated protein highly expressed in the central nervous system (CNS) [1,2]. Tau proteins are soluble, typically lack secondary structure, and qualify as intrinsically disordered [3,4]. These properties permit tau to carry out its fundamental functions inside neurons, such as promoting microtubule (MT) formation and regulating MT dynamics. Tau’s phosphorylation state is dynamic and contributes to its biological function. In the adult CNS, tau proteins range in size from 352 to 441 amino acids depending on the alterative splicing of exons 2, 3, and 10 [5]. Tau protein expression is mostly observed in neurons [6], but has also been detected in glia such as oligodendrocytes and astrocytes [7,8].

While tau’s unstructured properties are concomitant with its soluble state, under pathological conditions, ordered filaments composed of hyperphosphorylated tau accumulate into fibrils, which are present in clinically heterogeneous diseases known as tauopathies [9]. These diseases include Alzheimer’s disease (AD), Pick’s disease (PiD), progressive supranuclear palsy (PSP), and corticobasal degeneration (CBD) [10], which are distinguished by their clinical, histological, and biochemical profiles. Clinically, AD manifests as cognitive deficits associated with an impairment in performing daily activities; these symptoms can present as amnestic or non-amnestic [11]. PiD, PSP, and CBD are types of frontotemporal lobar degeneration-tau (FTLD-tau) diseases [12]. Neuropathologically confirmed PiD patients have been noted to exhibit behavioral and language abnormalities [13], while patients with PSP and CBD often present with motor impairments [14,15]. Interestingly, the neuropathological hallmarks of these tauopathies have characteristic morphology and isoform abundance [16]. These diseases also vary in their regional distribution of tau aggregates in the brain, which helps explain the heterogeneity in clinical symptoms observed in patients.

There are over 80 potential phosphorylation sites found on the longest (2N/4R) tau isoform expressed in the human CNS, several of which have been widely studied using phosphorylation-dependent tau antibodies. Many of these phosphorylation sites are found in serine/threonine-proline motifs found in the proline-rich region (PRR) of tau that are phosphorylated by proline-directed kinases [17]. This region is of particular interest due to the abundance of sites available and occupied by phosphate groups in sarkosyl-insoluble fractions isolated from individuals with AD [18]. Notably, the phosphorylation states of several sites within this region, such as Thr181, Thr205, and Thr217, have been shown to be biomarkers for AD found in cerebrospinal fluid (CSF) and plasma [19,20,21].

In this study, we have characterized two monoclonal tau antibodies raised against a peptide in the PRR (208–225) with phosphorylation sites at Thr212, Ser214, Thr217. These antibodies (which we refer to as 5E2 and 2F12) have demonstrated enhanced signal when sites T212, S214, and T217 are phosphorylated, but are not completely dependent on phosphate presence. By biochemical methods, we observed that these antibodies are dependent upon conformational epitopes modulated by adjacent phosphorylation sites. Additionally, these antibodies label the neuropathological hallmarks of AD, PiD, CBD, and PSP. These antibodies will provide an effective tool for studying the biophysical properties of tau inclusions and their pathogenesis in associated tauopathies.

## 2. Results

### 2.1. Epitope Characterization of Novel Tau Antibodies 5E2 and 2F12 by ELISA

**Figure 1:**
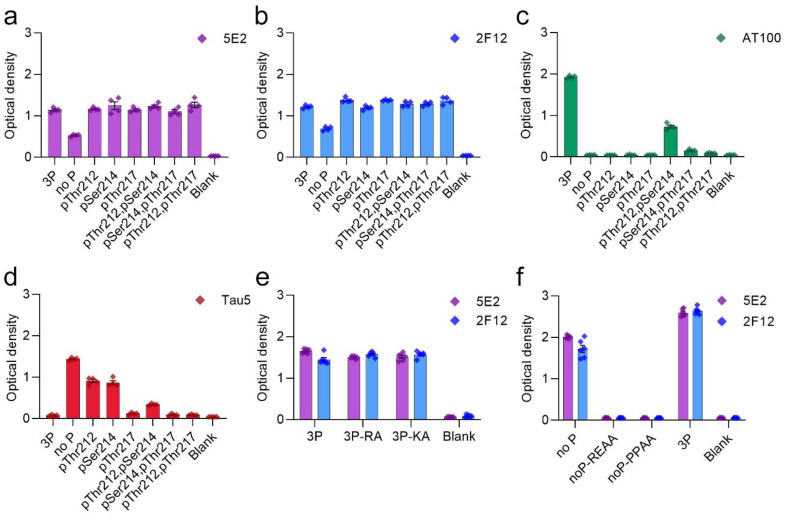
Epitope characterization of 5E2 and 2F12 antibodies with phospho- and mutated peptides by ELISA. Synthesized peptides are described in Table 1. Antibody binding of 5E2 (**a**), 2F12 (**b**), AT100 (**c**), and Tau5 (**d**) were investigated by ELISA as with immunization peptide (3P), phospho-peptide permutations, and non-phosphorylated tau peptide (no P). Further epitope characterization of 5E2 and 2F12 antibodies was conducted using alanine mutated peptides (**e**,**f**). Numbering of amino acid residues is according to the longest tau isoform present in the CNS.

**Figure 2:**
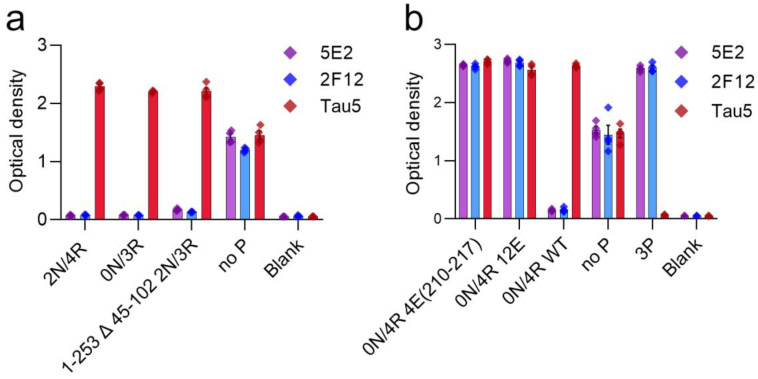
Epitope characterization of 5E2 and 2F12 using recombinant tau proteins by ELISA. Characterization of 5E2 and 2F12 epitope was further investigated by ELISA using full-length tau isoforms (2N/4R, 0N/3R), truncated tau protein 1–253 Δ 45–102 of 2N/3R, and no P peptide (**a**). Phospho-mimetic tau proteins (0N/4R 4E(210–217), 0N/4R 12E), WT 0N/4R as well as no P and 3P peptides were used for additional characterizations (**b**). Numbering is according to the longest tau isoform present in the CNS.

Tau antibodies 5E2 and 2F12 were characterized by ELISA using peptides (Table 1) and recombinant human tau proteins. We initially performed ELISA with 5E2, 2F12, and AT100, which is a positive control that reportedly recognizes tau phosphorylated at both Thr212 and Ser214 [22]. Both 5E2 and 2F12 antibodies recognized the immunization peptide, termed 3P, and the same peptide sequence, but with various phosphorylation permutations of Thr212, Ser214 and Thr217 (Figure 1a,b). 5E2 and 2F12 also recognized the non-phosphorylated version of the immunization peptide, termed no P, but the signals were less compared to phospho-peptides (Figure 1a,b). AT100 reacted with the peptide phosphorylated at both Thr212 and Ser214, but not with the single phospho-peptides or the other dual phospho-peptides, and interestingly, the signal was also enhanced when all three residues Thr212, Ser214, and Thr217 were phosphorylated (Figure 1c). Since binding for both 5E2 and 2F12 was not completely dependent upon phosphorylation, the epitope must be shared among all peptides. The phosphorylation-independent epitope for the monoclonal antibody Tau5 has been reported to be residues 218–225 [23], which is shared between the immunization peptide and the other peptide permutations used here. Therefore, we sought to investigate Tau5 binding with these peptides by ELISA and found that Tau5 does bind to the no P peptide (Figure 1d). We also found that Tau5 binding is influenced by adjacent phosphorylation sites and signal is drastically reduced as sites adjacent to the epitope were phosphorylated. Notably, Tau5 signal by ELISA is most influenced by the phosphorylation of Thr217. To further map the epitope for 5E2 and 2F12, we used peptides with alanine mutations at either the amino (N) and carboxyl (C) regions of the 3P peptide (Table 1). Alanine mutations at either the far N terminus (3P-RA) or the far C terminus of the 3P peptide (3P-KA) did not influence binding of 5E2 and 2F12 (Figure 1e). We next used alanine mutations within the no P peptide at Pro218/Pro219 (PPAA) and Arg221/Glu222 (REAA). While antibody binding of no P and 3P peptides was observed, both 5E2 and 2F12 antibody binding of peptides noP-PPAA and noP-REAA was not present (Figure 1f), indicating that these residues (Pro218-Glu222) are within the epitope of the 5E2 and 2F12 antibodies. We next investigated the ability of these antibodies to recognize recombinant full-length forms of tau expressed in bacteria that are not phosphorylated. We used the longest (2N/4R) and shortest (0N/3R) human tau isoforms that are normally expressed in human brains. Surprisingly, while both 5E2 and 2F12 were capable of binding to the no P peptide, which shared sequence identity with these recombinant proteins, neither antibody was reactive towards tau isoforms (Figure 2a). They also did not react with a shorter C-terminal truncated version of 0N/3R ending at residue 195 that is equivalent to 1–253 Δ 45–102 of 2N/3R tau (Figure 2a).

We next investigated antibody binding in the context of phospho-mimetics within 0N/4R tau (Table 2) to determine if the presence of charged glutamates within the region of the immunization peptide would influence antibody binding. Antibody binding was restored by these charged residues, as shown by ELISA with tau 0N/4R 4E(210–217) and tau 0N/4R 12E compared to a lack of signal, which was observed with 0N/4R WT (Figure 2b). Additionally, although Tau5 binding was reduced by phosphorylation at Thr212 and Ser214, and even more so at Thr217 (Figure 1d), glutamate phospho-mimetics at these residues did not affect Tau5 binding (Figure 2b).

### 2.2. 5E2 and 2F12 Immunoreactivity Is Dependent on the Phosphorylation State Adjacent to the Core Epitope and Enhanced by Tau Pathogenic Mutants in HEK293T Cells

**Figure 3:**
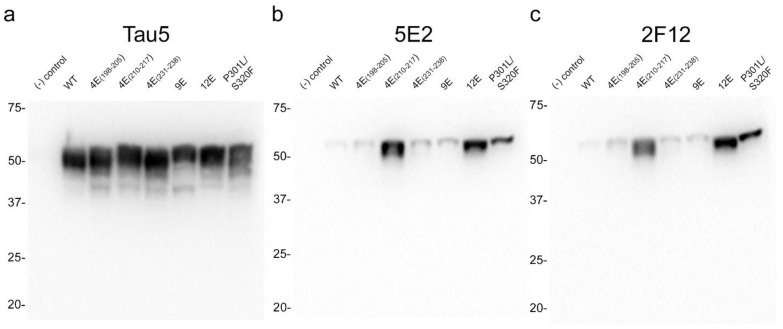
Epitope characterization of 5E2 and 2F12 antibodies using wild-type, mutant and phospho-mimetic tau proteins expressed in HEK293T cells. Tau5 (**a**), 5E2 (**b**), and 2F12 (**c**) immunoblotting labelling of HEK293T cells lysates expressing wild-type (WT), phospho-mimetics, and P301L/S320F tau proteins. Relative mobilities of molecular-weight markers are indicated on the left side of each blot. Numbering is according to the longest tau isoform present in the CNS.

The properties of the 5E2 and 2F12 antibodies were further characterized by expressing WT tau and phospho-mimetic constructs (Table 2), with residues altered both upstream and downstream of the core epitopes in HEK293T cells followed by immunoblotting analysis (Figure 3). The presence of nine phospho-mimetic residues in the carboxy-terminal region, termed 9E, did not influence 5E2 and 2F12 binding. The presence of 12 phospho-mimetic residues just upstream of the core epitope region termed 12E greatly increased 5E2 and 2F12 binding. Breaking down these 12 phospho-mimetic residues into a series of four mutations termed 4E(198–205), 4E(231–238), and 4E(210–217) revealed that this enhanced binding is due to the latter set of four phospho-mimetic residues. Furthermore, despite not containing phospho-mimetics at these sites, pro-aggregant P301L/S320F tau showed enhanced signal compared to WT 0N/4R. This increase could be due to the P301L/S320F mutations promoting conformation changes within tau or indirectly due to robust aggregation that also results in tau hyperphosphorylation, which can occur in HEK293T cells [24,25]. The signal ratio of 4E(210–217) tau/WT tau is 36.1 for 5E2 and 34.5 for 2F12, the signal ratio of 12E tau/WT tau is 30.7 for 5E2 and 39.9 for 2F12, while the signal ratio of P301L/S320F tau to WT tau is 13.4 for 5E2 and 25 for 2F12.

### 2.3. Immunohistochemical and Biochemical Characterization of 5E2 and 2F12 Antibodies Using Mice

**Figure 4:**
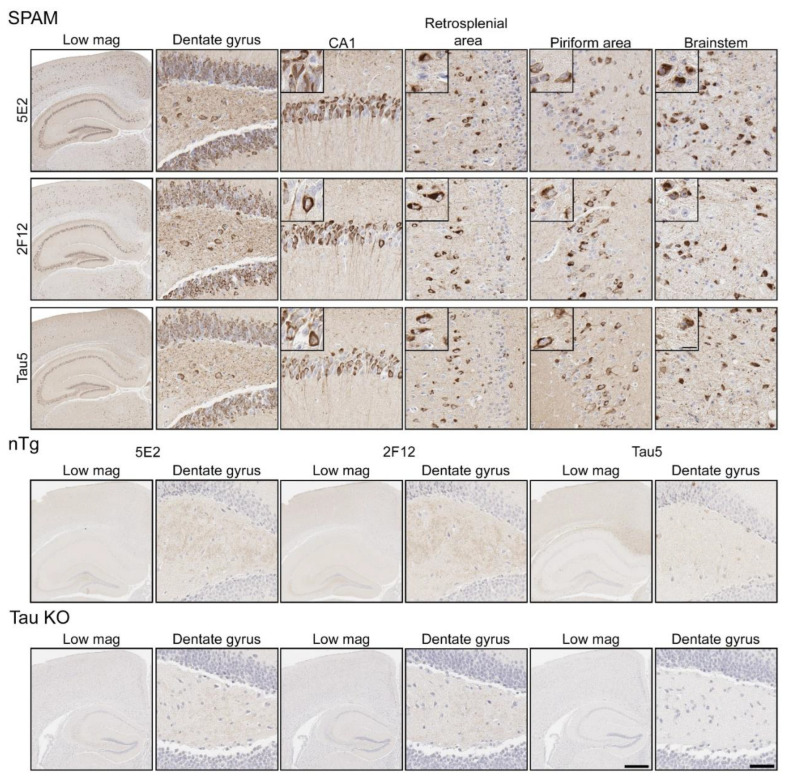
Immunohistochemical labelling of tau neuropathology in a mouse model of tauopathy with novel antibodies 5E2 and 2F12. Mouse brain tissue from a mouse model of tauopathy (SPAM), non-transgenic (nTg), and a tau null (Tau KO) mice was immunostained with 5E2, 2F12, and Tau5. An N of 9–11 SPAM and nTg mice were stained with 5E2 and 2F12. Scale bar for insets is 15 µm. Scale bar of high-magnification images is 50 µm. Scale bar of low-magnification images is 500 µm.

**Figure 5:**
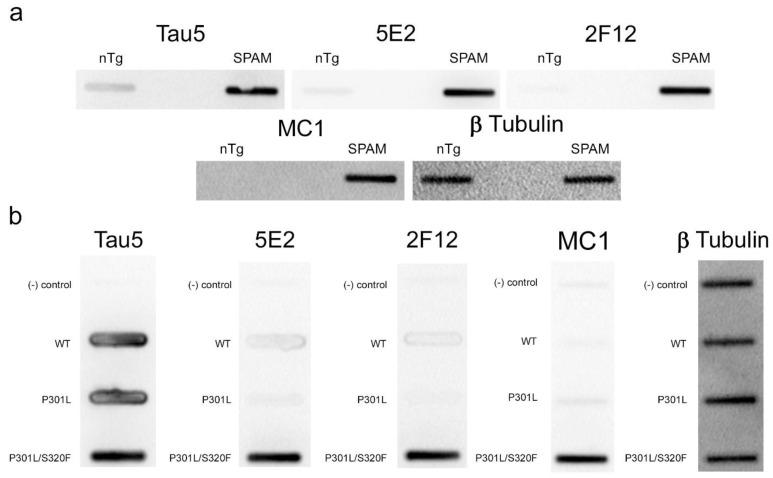
5E2 and 2F12 immunolabelling under non-denaturing conditions. Non-denaturing immunoblotting using a slot blot assay was performed on mouse and HEK293T cell lysates. Antibody labelling was investigated using brain lysate from nTg and SPAM Tg mice (**a**) and lysates from non-transfected HEK293T cells (−) and HEK293T cells transfected to express WT, P301L or P301L/S320F 0N/4R tau (**b**). Several antibodies were used as controls: Tau5 as control for mouse and human tau, MC1 as conformation-dependent tau antibody, and anti-β-tubulin as a loading control.

Immunohistochemistry (IHC) was performed using mouse brain tissue from tau KO [26], non-transgenic (nTg), and pathogenic P301S/S320F 0N/4R tau (SPAM) transgenic mice [25]. Notably, SPAM mice developed neuropathology throughout the cerebral cortex and hippocampus. By IHC, both 5E2 and 2F12 labelled abundant neurofibrillary tangle (NFT)-like inclusions in several areas of the brain (Figure 4). Tau5, used as a positive control, also labelled these inclusions. In non-transgenic (nTg) and tau null (Tau KO) mice, both 5E2 and 2F12 only showed some faint staining, such as within hippocampal subregions. Tau5, which also labels mouse tau, showed staining in nTg brain tissue, but did not show staining in Tau KO brain (Figure 4). We also performed a peptide pre-absorption experiment with the 3P peptide and 5E2 and 2F12. 5E2 and 2F12 without incubation with 3P peptide, which showed strong immunostaining of NFT-like inclusions in SPAM mice as expected, while incubation with the 3P peptide showed a reduction in immunostaining of SPAM mice by IHC (Supplementary Figure S1). We next performed immunoblotting with brain lysate from SPAM Tg, nTg, and Tau KO mice (Supplementary Figure S2). By immunoblotting under denaturing conditions, as expected, Tau5 detected tau in both nTg and SPAM Tg brain tissue, while 5E2 and 2F12 detected hyperphosphorylated tau in SPAM Tg brain tissue as observed by shift in mobility. AT100 also detected hyperphosphorylated tau in SPAM Tg brain lysate. It should be noted that 5E2, 2F12, and AT100 appear to not be completely tau-specific in mouse brain, as observed by a low-molecular-weight protein (~18 kD) detected by 5E2 and 2F12 in tau KO lysate and ~80 kD and ~30 kD proteins detected by AT100 in tau KO lysate (Supplemental Figure S2). While 5E2 and 2F12 bound to pathogenic tau expressed in SPAM mice, as observed by IHC (Figure 4), under immunoblotting denaturing conditions the signal was relatively weak for pathological tau (Supplemental Figure S2), which suggests that the epitope preserved in fixed tissue is disrupted by conditions required for SDS-PAGE. Therefore, we performed slot blot assays, which do not require denaturing conditions (Figure 5). Using nTg and SPAM Tg brain lysate, Tau5, 5E2 and 2F12 all showed enhanced immunoreactivity in SPAM Tg lysate that was much stronger for 5E2 and 2F12 compared to SDS-PAGE. MC1 conformation-dependent tau antibody also preferentially immunolabelled SPAM Tg lysate compared to nTg brain lysate (Figure 5a). β-tubulin was used as a loading control.

To further confirm that the 5E2 and 2F12 epitopes are enhanced by tau aggregation in mammalian cells, WT, P301L, and P301L/S320F tau proteins were expressed in HEK293T cells and analyzed by slot blotting (Figure 5b). WT, P301L, and P301L/S320F tau were present at similar levels as shown with Tau5 antibody. Much higher signal was observed specifically for pro-aggregant P301L/S320F tau with 5E2, 2F12, and MC1 antibodies. β- tubulin was used as a loading control.

### 2.4. 5E2 and 2F12 Labelling of Tau Neuropathology in Alzheimer’s Disease and Primary Tauopathies

**Figure 6:**
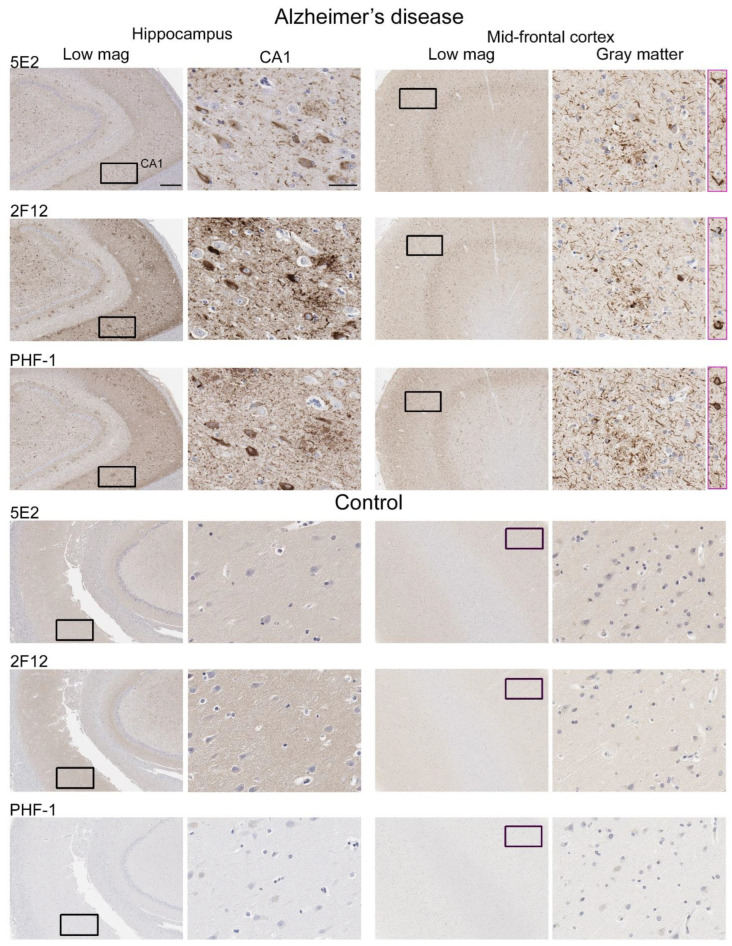
Immunohistochemical labelling of tau neuropathology in Alzheimer’s disease brain tissue using novel antibodies 5E2 and 2F12. Hippocampus and mid-frontal cortex were investigated in both Alzheimer’s disease and control brain tissue using novel antibodies 5E2 and 2F12, as well as phospho-dependent (pS396/pS404) tau antibody PHF-1. For AD cases, hippocampal tissue from case AD-8 and mid-frontal cortex tissue from case AD-5 are shown. For control cases, hippocampal tissue from case control-8 and mid-frontal cortex tissue from control-3 is shown. Low-magnification images of CA1 within the hippocampus and gray matter of the mid-frontal cortex are indicated. Black boxes in low-magnification images indicate general area in which the high-magnification image is from, high magnification images were cropped from the larger image where black box is located. Pink boxes are from different areas within corresponding low-magnification images. Scale bar for high-magnification images is 50 µm. Scale bar for low-magnification images is 500 µm.

**Figure 7:**
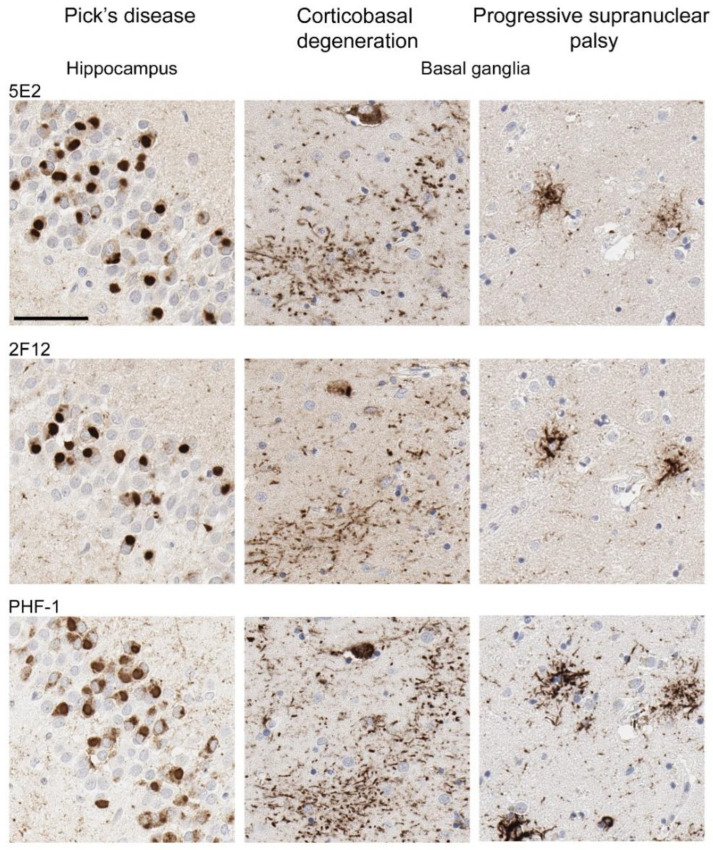
Immunohistochemical labelling of tau pathology in primary tauopathies. The hippocampus for Pick’s disease cases and the basal ganglia (striatum) for corticobasal degeneration and progressive supranuclear palsy cases were investigated for the presence of tau neuropathological lesions using novel antibodies 5E2 and 2F12, as well as phospho-dependent tau antibody PHF-1. For PiD, case PiD-1 is shown. For CBD, case CBD-4 is shown. For PSP, case PSP-6 is shown. Scale bar is 60 µm.

Neuropathological tau burden varies between tauopathies, and often, cell types affected and regional differences are observed in postmortem tissue. We examined the hippocampus and mid-frontal cortex in neuropathologically confirmed cases of AD (Figure 6). We observed that both 5E2 and 2F12 robustly labelled the neuropathological hallmarks of AD, including neurofibrillary tangles, neuropil threads, and neuritic plaques within both of these regions. PHF-1, a tau phosphorylation-specific antibody to Ser396/Ser404 [27] also labelled these pathological inclusions as a positive control (Figure 6). We also observed some weaker neuropil immunostaining of the gray matter, with both 5E2 and 2F12 tau antibodies in both the hippocampus and mid-frontal cortex of control individuals consistent with previous studies with other tau antibodies [28]. In controls, PHF-1 immunoreactivity was minimal, although some immunostaining within the cell body was observed (Figure 6). Rare neuropil threads and very few tangles were observed in the hippocampus of control cases, unsurprising due to these cases having low Braak stages (Table 3). Immunolabelling of 5E2 and 2F12 was also investigated in neuropathologically diagnosed individuals with PiD, CBD, and PSP (Figure 7). Within the granule layer of the dentate gyrus of PiD cases, 5E2 and 2F12 robustly labelled Pick bodies, while 5E2 and 2F12 antibodies also strongly revealed astrocytic plaques in CBD cases and tufted astrocytes in PSP cases (Figure 7). We also performed semiquantitative analysis of frontal cortex from AD and control patients as well as striatum from CBD, PSP, and control patients, which showed that all cases were positively stained with 5E2 and 2F12 as well as PHF-1. These new antibodies displayed similar specificity to PHF-1 for AD, CBD, PSP, and control cases for the brain regions investigated. However, sensitivity was modestly lower for PSP pathology (Supplemental Figure S3).

## 3. Discussion

Pathological tau is characterized by detergent insolubility [29,30,31], decreased mobility by SDS-PAGE due to phosphorylation events [9,32], reduced ability to promote microtubule polymerization [32,33], and conformational changes that manifest as filamentous aggregates found in neurons and glial cells in the brains of individuals with neuropathological diagnosis of a tauopathy [34,35,36,37]. While tau proteins are observed to be phosphorylated physiologically [38], during disease, hyperphosphorylated tau proteins are observed as the neuropathological hallmarks of these tauopathies [12,39], but the sequence of events which leads to these fibrillar lesions is not completely understood.

The tau molecule can be divided into the projection domain and the assembly domain [40]. Within these domains is the PRR, which contains many Ser/Thr sites phosphorylated in AD [41] and has also been implicated in facilitating microtubule assembly [40,42,43,44]. Many available antibodies are directed to phosphorylation sites within the PRR, which allows the study of their neuropathological signatures. Likewise, much work has been done to determine phosphorylation sites within tau that are more pathological in nature and those that distinguish between tauopathies. Several sites have been identified and shown to be elevated, such as pThr181 and pThr217 in brain tissue, cerebrospinal fluid (CSF), and plasma [21,45,46,47,48,49]. While few neuropathological investigations of the pThr217 site have been conducted, the pThr217 site has been shown to differentiate AD from other neurodegenerative diseases [21,50] and investigation of pThr217/Thr217 has revealed high correlation with tau and amyloid positron emission tomography, more so than other measures using immunoassays as well as mass spectrometry [19], which led to our interest in this site.

In our study, we generated two monoclonal antibodies, 5E2 and 2F12, which were raised against a peptide with triple phospho-sites at Thr212/Ser214/Thr217. Using ELISA characterization, we discovered the epitope consisted of amino acids Pro218-Glu222 (Figure 1) which did not require phosphorylation presence, but signal was enhanced when at least one phosphate was present. Surprisingly, while signal was observed with the non-phosphorylated peptide, a signal was not detected with native tau isoforms, despite shared sequence identity (Figure 2).

**Figure 8:**
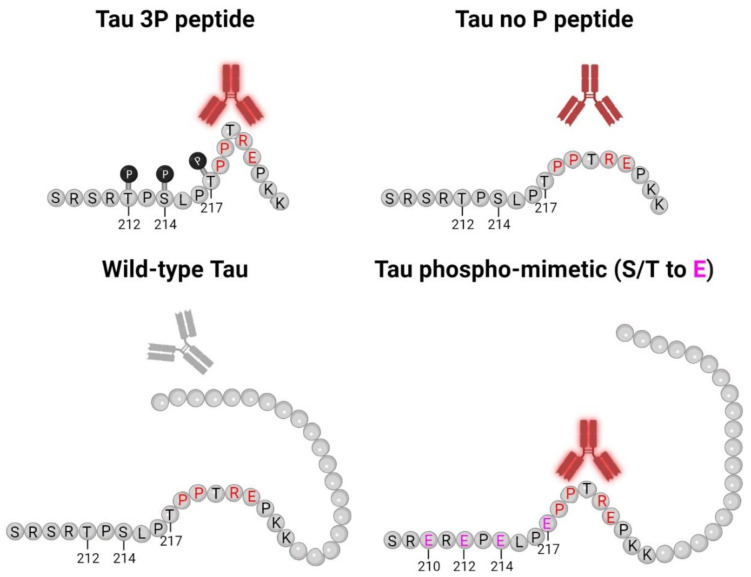
5E2 and 2F12 Tau binding schematic. This model for 5E2 and 2F12 antibody binding shows that both antibodies exhibit strong immunoreactivity for the phosphorylated tau peptide, while the signal for the non-phosphorylated tau peptide is weaker, but still present. Both antibodies require Pro218-Glu222 for binding (residues highlighted in red), which likely contribute to the conformational character of the epitope. While Pro218-Glu222 is also present in full-length wild-type tau, the epitope for 5E2 and 2F12 is masked due to the adjacent tau sequence, which is lacking for the non-phosphorylated tau peptide, allowing for the antibodies to bind. The introduction of negative charges due to phospho-mimetics at residues 210, 212, 214, 217 (highlighted in pink) unmasks the epitope, allowing for 5E2 and 2F12 to bind. Our data collectively show that both 5E2 and 2F12 rely on a conformational epitope, which is indicated by the bend in the peptides and proteins. Since tau antibodies show stronger immunoreactivity for the phosphorylated peptide compared to the non-phosphorylated peptide, the presence of these phosphorylation sites, which are elevated in human AD brain tissue, also likely contribute to the conformational characteristic of these antibodies. Created with Biorender.com.

Early investigation of tau proteins found that tau is highly soluble, heat stable, and possesses little secondary structure [2,3,51]. Further studies also observed the intrinsically disordered nature of tau protein [4,52], which allows for the adoption of a cornucopia of conformations. Our data shows that both 5E2 and 2F12 are dependent upon a conformation-specific epitope. While binding of native tau was not observed, in the presence of glutamate mutations adjacent to the epitope, which mimic phosphorylation sites, binding was similar to the immunization peptide (Figure 2). These data suggest that the 5E2 and 2F12 epitopes are hidden in native tau, but in the presence of negative charges, the tau molecule is opened up, allowing for binding of 5E2 and 2F12 to the exposed epitope (Figure 8). Additionally, the transfection and subsequent immunoblotting of tau phospho-mimetics and pro-aggregant P301L/S320F tau proteins with 5E2 and 2F12 showed that the epitope can be recreated by the adjacent phospho-mimetic sites or a stable conformation from the P301L/S320F tau protein (Figure 3). The conformational character of the epitope is also observed by the denaturing and non-denaturing immunoblotting data. By IHC with 5E2 and 2F12, we observed robust staining in our mouse model of tauopathy of NFT-like inclusions throughout the cerebral cortex and hippocampus (Figure 4), two areas highly affected during the course of AD according to Braak staging [53]. Interestingly, compared to our IHC data, we observed low signal on western blotting (Supplementary Figure S2), but upon non-denaturing immunoblotting, immunoreactivity was readily observed in brain lysate from our mouse model of tauopathy (Figure 5a), suggesting the conformational epitope was disrupted by conditions required for SDS-PAGE, which has previously been reported with other conformation-specific tau antibodies using heat and chemical denaturants [54,55,56]. Similar to Western blotting, pro-aggregant P301L/S320F tau was highly immunoreactive with 5E2 and 2F12 compared to WT tau by non-denaturing immunoblotting, suggesting the immunolabelling of a stable conformation (Figure 5b). By Western blotting, low-molecular-weight nonspecific bands were also detected using 5E2 and 2F12, which perhaps can be reduced by removing endogenous IgGs or using secondary antibodies that target light chains of the antibody protein, which has previously been accomplished to reduce nonspecificity [57].

Distinguishing normal and pathological phosphorylation events has been mediated through the use of mass spectrometry and monoclonal antibody studies [18,27,39,58,59,60,61,62,63,64,65,66], while the identification of conformational events leading to fibril formation has been more cumbersome to study. Although tau largely lacks secondary structural elements, global conformations have been detected known as the “paper clip” fold where the C-terminus and N-terminus fold onto the microtubule-binding domain [67]. Monoclonal antibodies such as MC1, Alz-50, and Tau-66 epitopes are formed by a discontinuous tau sequence, where—although not phosphorylation-dependent since epitopes were characterized using recombinant protein [54,68]—it has been observed that the paperclip fold is influenced by phosphorylation sites within the PRR and the C-terminus that facilitate the formation the MC1 epitope [69]. Additionally, it has been shown that the MC1 epitope is an early signature of tau conformation that may come before the development of tau filaments [70], suggesting that targeting tau conformations may be viable therapeutic targets.

Both 5E2 and 2F12 are not conformation-dependent due to a discontinuous epitope such as other conformation-dependent antibodies MC1, Alz-50, Tau-66, SKT82, and DMR7 [54,55,68], but rely on conformation(s) within a limited peptide sequence similar to what has been previously observed with the TG3 antibody [71,72]. TG3 is a monoclonal antibody that labels tau neuropathology in AD cases [72] that is specific for phosphorylation at Thr231 and was shown to have enhanced signal when halogenated alcohol, trifluoroethanol, was used instead of water during peptide coating [71]. Tau peptide coated in trifluoroethanol has been shown to promote antibody binding due to the increased presence of secondary structural elements such as β-sheets or turns determined by ELISA and circular dichroism [73]. Perhaps the phosphates within the immunization peptide serve a similar purpose in which 5E2 and 2F12 antibodies can bind to certain conformations within the peptides, which may be further exposed when phosphates are present, which results in enhanced antibody binding (Figure 8). Likewise, using nuclear magnetic resonance, several polyproline II helices have been observed within the proline-rich region, specifically within residues Pro216–Pro223 [74], where the epitope for 5E2 and 2F12 lies, which may potentially act as another structural element contributing to antibody binding.

Both 5E2 and 2F12 label the neuropathological hallmarks of AD, PiD, CBD, and PSP (Figure 6 and Figure 7), suggesting similar molecular mechanisms may contribute to the formation of this epitope across several tauopathies. Monoclonal antibodies 5E2 and 2F12 will serve as novel tools to investigate conformational events modulated by phosphorylation sites within the tau protein. The ability to study the conformations that precede tau amyloids found in neuropathological inclusions will be useful to determine potential biomarkers and examine the relationship between phosphorylation and conformation, as these two tau modifications are highly associated with neuropathological features of tauopathies.

## 4. Materials and Methods

### 4.1. Antibodies and Peptides

See Table 1 for a complete list of all synthetic peptides used. These were synthesized and purified as a service provided by GenScript (Piscataway, NJ, USA). Mouse monoclonal anti-tau antibody AT100 specific for tau phosphorylated at both Thr212 and Ser214 [22] was purchased from Thermo Fisher (Waltham, MA, USA). Tau 5 (Thermo Fisher Scientific, Waltham, MA, USA) is mouse monoclonal antibody with an epitope including residues 218–225 in human tau [23]. MC1 is a conformation-dependent mouse monoclonal antibody [68] that was a generous gift from the late Dr. Peter Davies. A mouse monoclonal anti-actin antibody (clone C4, Thermo Fisher Scientific, Waltham, MA, USA) and a mouse anti β-tubulin antibody (Clone TUB2.1, Sigma Aldrich, St.Louis, MO, USA) were also used. Antibody isotypes for 5E2 and 2F12 were determined to be IgG_1_ using a mouse monoclonal isotyping kit (Millipore Sigma, Burlington, MA, USA).

### 4.2. Generation of New Tau Monoclonal Antibodies 5E2 and 2F12

A synthetic peptide (CSRSR(pThr)P(pSer)LP(pThr)PPTREPKK) corresponding to amino acids 208–225 in the 2N/4R human tau isoform with residues Thr212, Ser214 and Thr217 phosphorylated was used for immunization. This peptide was chosen as it contains all 3 phosphorylated amino acid residues with additional adjacent residues allowing for appropriate size for the immunization peptide. The peptide included an added Cys residue at the amino-terminus that allowed for conjugation to inject maleimide-activated mariculture keyhole limpet hemocyanin (mcKLH) (Thermo Scientific, Waltham, MA, USA). All procedures were performed according to the NIH Guide for the Care and Use of Experimental Animals and were approved by the University of Florida Institutional Animal Care and Use Committee. The peptide–KLH conjugate was used to immunize female BALB/c mice (Jackson Laboratory, Bar Harbor, ME, USA) as previously described [75]. Spleens from the mice were harvested, and the white blood cells were fused with mouse myeloma cells (Sp2/O-Ag14; ATCC, Manassas, VA, USA). Hybridoma clones were selected using HAT supplement (Sigma Aldrich, St. Louis, MO, USA) and the surviving clones were initially screened for reactivity by enzyme-linked immunosorbent assay (ELISA) using the immunization peptide, but further assessed using a series of peptides and recombinant tau proteins, as described below.

### 4.3. ELISA

ELISA plates (96-well, Thermo Fisher, Waltham, MA, USA) were coated with 100 ng of peptides or recombinant tau proteins in 100 µL phosphate-buffered saline (PBS) for peptides or H_2_O for proteins per well. Wells were washed with PBS and blocked with 5% FBS/PBS. Primary antibodies were added to blocking solution and incubated at room temperature. After PBS washes, plates were incubated with goat anti-mouse IgG + IgM (H + L) horseradish peroxidase (HRP)-conjugated antibody (Jackson Immuno Research Labs, West Grove, PA, USA) in 5% FBS/PBS. Plates were washed with PBS and 3,3’,5,5’-tetramethylbenzidine (TMB substrate, Thermo Fisher Scientific, Waltham, MA, USA) was added to each well. The reactions were stopped by adding HCl and the optical density was measured at 450 nm with a plate reader.

### 4.4. Generation of Phospho-Mimetic Constructs

Human tau phospho-mimetic constructs in the pcDNA3.1 vector were generated as a service by GenScript (Piscataway, NJ, USA). 12E 0N/4R tau is mutated at residues Ser198, Ser199, Ser202, Thr205, Ser210, Thr212, Ser214, Thr217, Thr231, Ser235, Ser237, and Ser238 to glutamate residues (E); 4E(198–205) 0N/4R tau were mutated at Ser198, Ser199, Ser202, and Thr205 to glutamate residues (E); 4E(210–217) 0N/4R tau were mutated at Ser210, Thr212, Ser214, and Thr217 to glutamate (E); 4E(231–238) 0N/4R tau were mutated at Thr231, Ser235, Ser237, and Ser238 to glutamate residues (E); 9E 0N/4R tau contained residues mutated at Tyr394, Ser396, Ser400, Thr403, Ser404, Ser409, Ser412, Ser422, and Ser435 to glutamate residues (E). Sanger sequencing was performed on all constructs.

### 4.5. Recombinant Tau Protein Expression and Purification

Human tau isoforms 2N/4R, 0N/4R, and 0N/3R, and truncated tau protein 1–253 Δ 45–102 of 2N/3R, and human tau 0N/4R phospho-mimetic (12E and 4E(210–217)) constructs were expressed from the bacterial plasmid pRK172 [76] in BL21 (DE3) *Escherichia coli* (Agilent Technologies, Santa Clara, CA, USA) and purified as previously described [24,77]. Protein concentration was measured by bicinchoninic acid (BCA) assay with bovine serum albumin (BSA) as a standard.

### 4.6. HEK293T Cells and Calcium Phosphate Transfection

HEK293T cells were cultured in Dulbecco’s modified Eagle medium-high glucose (4.5 g/mL) supplemented with 10% fetal bovine serum (FBS), 100 U/mL penicillin, and 100 µg/mL streptomycin at 37 °C and 5% CO_2_. Cells were transfected using calcium phosphate as previously described [78].

### 4.7. Histological and Biochemical Analysis of Mouse Tissue

Mice were humanely euthanized in a CO_2_ chamber and were perfused with a heparin–PBS solution. For IHC, brains were fixed in 70% ethanol/150 mM solution, embedded in paraffin, and sectioned at 5 µm. For biochemical analysis, brain tissue from SPAM (S320F/P301S 0N/4R tau) transgenic [25], non-transgenic (nTg), and tau-null mice [26] brain tissue was isolated and frozen. For Western blotting, mouse tissue was sonicated in 4% SDS/50mM Tris, pH7.5 and heated for 10 min at 95 °C. Following determination of protein concentrations with the BCA assay with BSA as a standard, 5× sample buffer was added to a final 1× concentration (10 mM Tris, pH 6.8, 1 mM EDTA, 40 mM DTT, 0.005% bromophenol blue, 0.0025% pyronin yellow, 1% SDS, 10% sucrose) and samples were heated for 10 min at 95 °C, and stored at −80 °C until further use.

### 4.8. Western Blotting Analysis

Mouse brain lysate samples (10 µg) were loaded on 10% SDS–polyacrylamide gels and resolved by SDS-PAGE, and electrophoretically transferred onto 0.45 µm nitrocellulose membranes. Membranes were blocked in 5% powdered milk or 5% BSA in Tris-buffered saline (50 mM Tris pH 7.5, 150 mM NaCl; TBS) for 1 h and incubated overnight at 4 °C in primary antibodies diluted 1:1000 in 5% milk/TBS, 5% BSA/TBS or undiluted cell culture media for 5E2 and 2F12. Blots were washed 3 times in TBS incubated with goat anti-mouse IgG + IgM (H + L) secondary antibody conjugated with HRP (Jackson ImmunoResearch, West Grove, PA, USA) for 1 h at room temperature. Membranes were then washed in TBS and developed using Western Lightning Plus ECL reagents (PerkinElmer Life Sciences, Waltham, MA, USA) and imaged using chemiluminescence (PXi, Syngene, Frederick, MD, USA). Tau bands were measured using ImageJ software (version 1.53k).

### 4.9. Immunohistochemistry

Formalin fixed paraffin-embedded human brain from the University of Florida Neuromedicine Brain Bank and ethanol-fixed paraffin-embedded mouse tissue (see above) were used for histopathological analysis. Slides were deparaffinized in xylenes and rehydrated in a graded series of descending alcohols (100%, 100%, 90%, 70%) and then washed in water. Heat-induced epitope retrieval was performed using a steamer and a target retrieval solution of citrate pH 6 (Agilent Technologies, Santa Clara, CA, USA) for 1 h. Slides were then allowed to cool in running water. Endogenous peroxidases were quenched using 1.5% H_2_O_2_ with 0.005% triton in PBS for 15–20 min. Slides were thoroughly washed in water and then washed once in 0.1 M Tris, pH 7.6 for 5 min. A 2% FBS/0.1 M Tris, pH 7.6 solution was used as blocking for at least 5 min at room temperature (RT). Antibodies 5E2 and 2F12 were applied to tissue undiluted, Tau5 was diluted 1:5000 and PHF-1 was diluted 1:1000 in blocking solution and incubated overnight at 4 °C. The next day, slides were washed 3 times in 0.1 M Tris, pH 7.6. Blocking solution was applied to slides for at least 5 min. For human tissue, horse anti-mouse IgG ImmPRESS conjugated to horse radish peroxidase (HRP) (Vector Laboratories, Newark, CA, USA) was used 1:10 (ImmPRESS:blocking) in combination with biotinylated anti-mouse IgG(H + L) (Vector Laboratories, Newark, CA) diluted 1:3000 in blocking solution for 1 h at RT. For mouse tissue, biotinylated anti-mouse IgG(H + L) diluted 1:3000 was applied to slides for 1 h at RT. Slides were subsequently washed 3 times in 0.1 M Tris, pH 7.6 and blocking solution was applied to slides for at least 5 min. An avidin–biotin complex (ABC) solution (Vectastain ABC Elite kit; Vector Laboratories, Newark, CA, USA) was diluted 1:3000 in blocking solution and applied to slides for 1 h at RT. Slides were washed 3x in 0.1 M Tris, pH 7.6 and tissue sections were developed using 3,3′-diaminobenzidine (DAB kit, KPL, Gaithersburg, MD, USA) and nuclei were counterstained using hematoxylin (Sigma Aldrich, St. Louis, MO, USA). Sections were washed in tap water and dehydrated in an ascending series of alcohols (70%, 90%, 100%, 100%) and xylenes and coverslipped using Cytoseal mounting media.

### 4.10. Semiquantification of Tau Neuropathology Immunostaining with PHF-1, 5E2, and 2F12 in Human Brain Tissue

Slides were scanned using an Aperio AT2 slide scanner at 40× magnification (Aperio Technologies, Vista, CA, USA). For semiquantification of tau pathology stained by antibodies 5E2, 2F12, and PHF1, 3 fields were captured per stained tissue sections, which were then scored by two raters using a 0 (no pathology), 1 (low pathology), 2 (moderate pathology), or 3 (high pathology) scale. The scores were then averaged and graphed using GraphPad Prism.

### 4.11. 5E2 and 2F12 Pre-Absorption with 3P Peptide

Both 5E2 and 2F12 were diluted 1:50 in blocking buffer and aliquoted into two tubes. The 3P peptide (10 mg/mL) was diluted 1:100 in one tube of diluted antibody, while the other tube received no peptide as a positive control., Antibodies 5E2 and 2F12 with and without peptide were incubated with agitation for 3 h at room temperature and then applied to nTg and SPAM mouse tissue. IHC of mouse tissue was performed as described above.

### 4.12. Non-Denaturation Immunoblotting

HEK293T cell or mouse tissue were lysed in PBS with 0.2% or 1% Triton X-100, respectively. Debris were cleared by centrifugation at 17,000× *g* for 5 min, and 20 µg was applied to 0.2 µm nitrocellulose membranes (Bio-Rad Laboratories, Hercules, CA, USA) using a Minifold II Slot Blot System (Schleicher & Schuell Biosciences, Keene, NH, USA). Membranes were dried and developed as described above for Western blotting analysis.

## Figures and Tables

**Table 1:** Summary of peptides used in the study.

Peptide Name	Peptide Sequence	Additional Note
Tau 208–225 (3P)	CSRSR(pThr)P(pSer)LP(pThr)PPTREPKK	extra Cys at the N-terminus
Tau 208–225 (no P)	CSRSRTPSLPTPPTREPKK	extra Cys at the N-terminus
Tau 208–225 (pT212)	CSRSR(pThr)PSLPTPPTREPKK	extra Cys at the N-terminus
Tau 208–225 (pS214)	CSRSRTP(pSer)LPTPPTREPKK	extra Cys at the N-terminus
Tau 208–225 (pT217)	CSRSRTPSLP(pThr)PPTREPKK	extra Cys at the N-terminus
Tau 208–225 (pT212, pS214)	CSRSR(pThr)P(pSer)LPTPPTREPKK	extra Cys at the N-terminus
Tau 208–225 (pS214, pT217)	CSRSRTP(pSer)LP(pThr)PPTREPKK	extra Cys at the N-terminus
Tau 208–225 (pT212, pT217)	CSRSR(pThr)PSLP(pThr)PPTREPKK	extra Cys at the N-terminus
3P-RA	CSASA(pThr)P(pSer)LP(pThr)PPTREPKK	extra Cys at the N-terminus
3P-KA	CSRSR(pThr)P(pSer)LP(pThr)PPTREPAA	extra Cys at the N-terminus
no P-REAA	CSRSRTPSLPTPPTAAPKK	extra Cys at the N-terminus
no P-PPAA	CSRSRTPSLPTAATREPKK	extra Cys at the N-terminus

Numbering is according to the longest isoform in the CNS (2N/4R). “p” indicates phosphorylation. Peptides are described in the “Section 4”.

**Table 2:** Summary of tau proteins used in the study.

Protein Name	Tau Isoform	Mutations
4E(198–205)	0N/4R	S198E, S199E, S202E, T205E
4E(210–217)	0N/4R	S210E, T212E, S214E, T217E
4E(231–238)	0N/4R	T231E, S235E, S237E, S238E
12E	0N/4R	S198E, S199E, S202E, T205E, S210E, T212E, S214E, T217E, T231E, S235E, S237E, S238E
9E	0N/4R	Y394E, S396E, S400E, T403E, S404E, S409E, S412E, S422E, S435E

Numbering is according to the longest isoform in the CNS (2N/4R). Phospho-mimetic mutations were created by mutating serine (S), threonine (T), or tyrosine (Y) residues to glutamate (E) as described in the “Section 4”.

**Table 3:** Summary of cases used in the study.

Case Type	Clinicopathological Diagnosis	Primary Neuropathological Diagnosis	Secondary Neuropathological Diagnosis	Thal	Braak	CERAD	APOE	Sex	Age	Region(s) Investigated
PSP-1	PSP	PSP	AD intermediate	5	III	moderate	4/4	m	72	Striatum
PSP-2	PSP	PSP	PART	0	I	none	3/3	m	67	Striatum
PSP-3	PSP	PSP	AD low	3	I	none	3/3	f	77	Striatum
PSP-4	PSP	PSP	AD low	2	II	sparse	3/3	m	79	Striatum
PSP-5	PSP	PSP	AD low	3	0	none	3/3	m	69	Striatum
PSP-6	PSP	PSP	AD intermediate	3	III	sparse	2/3	f	74	Striatum
PSP-7	PSP	PSP	LATE Stage2	1	II	none	3/3	m	77	Striatum
PSP-8	PSP	PSP	AD low	3	I	none	3/3	m	78	Striatum
CBD-1	CBD	CBD	ARTAG	0	0	none	3/3	f	71	Striatum
CBD-2	CBD	CBD	AD low	1	II	none	3/3	f	73	Striatum
CBD-3	CBD	CBD	AD low	3	II	none	2/4	f	70	Striatum
CBD-4	CBD	CBD	AD low	1	II	none	2/3	m	69	Striatum
CBD-5	vascular dementia/AD	CBD	remote infarct in basal ganglia	0	II	none	3/3	m	82	Striatum
CBD-6	FTD/PPA	CBD	AD low	1	I	none	3/3	m	66	Striatum
PiD-1	FTLD	PiD	AD low	3	II	sparse	3/3	m	75	Hippocampus
PiD-2	PiD	PiD	AD intermediate	5	III	sparse	3/3	m	82	Hippocampus
AD-1	AD	AD high	CAA focal, mild	5	V	frequent	3/3	f	85	Hippocampus, Mid-frontal cortex
AD-2	AD	AD high	CAA focal, mild	5	V	frequent	3/3	f	83	Hippocampus
AD-3	dementia	AD high	LATE stage 2, hippocampal sclerosis	5	VI	frequent	3/3	f	77	Mid-frontal cortex
AD-4	AD	AD high	CAA widespread, mild to moderate	5	VI	frequent	3/3	m	78	Mid-frontal cortex
AD-5	AD	AD high	LBD amygdala predominant	5	VI	frequent	3/3	m	70	Mid-frontal cortex
AD-6	AD	AD high	CAA focal, mild to moderate	5	VI	frequent	3/3	f	79	Mid-frontal cortex
AD-7	AD	AD high	CAA widespread, mild to moderate	5	V	frequent	3/3	f	83	Mid-frontal cortex
AD-8	LBD	AD high	CAA focal, moderate	5	V	frequent	3/4	m	74	Hippocampus, Mid-frontal cortex
AD-9	AD	AD high	CAA, moderate, widespread	5	V	frequent	3/4	f	86	Mid-frontal cortex
AD-10	AD	AD high	CAA widespread, severe	5	V	frequent	3/4	f	72	Mid-frontal cortex
AD-11	AD	AD high	CAA widespread, mild to moderate	4	VI	frequent	3/4	f	82	Mid-frontal cortex
AD-12	vascular dementia, possibly mixed	AD high	CAA moderate to severe	4	VI	frequent	3/4	m	73	Mid-frontal cortex
AD-13	AD	AD high	CAA focal, moderate	4	V	frequent	3/4	f	85	Mid-frontal cortex
AD-14	AD	AD high	CAA widespread, mild to moderate	5	V	frequent	3/4	m	77	Mid-frontal cortex
AD-15	dementia	AD high	CAA widespread, moderate to severe	5	V	frequent	3/4	m	80	Mid-frontal cortex
AD-16	dementia	AD high	LBD diffuse neocortical	5	VI	frequent	3/4	f	84	Mid-frontal cortex
AD-17	AD	AD high	CAA widespread, moderate	4	VI	frequent	4/4	f	86	Mid-frontal cortex
AD-18	AD	AD high	CAA widespread, moderate	4	VI	frequent	4/4	m	75	Mid-frontal cortex
AD-19	AD	AD high	CAA widespread, moderate	5	VI	frequent	4/4	m	70	Mid-frontal cortex
AD-20	AD	AD high	CAA widespread, moderate	5	VI	frequent	4/4	m	72	Mid-frontal cortex
AD-21	AD	AD high	CAA widespread, mild	5	VI	frequent	4/4	f	76	Mid-frontal cortex
AD-22	AD	AD high	CAA widespread, severe	5	V	frequent	4/4	f	84	Mid-frontal cortex
Control-1		PART	subacute microinfarct corpus callosum	0	II	none	3/3	f	90	Mid-frontal cortex
Control-2		PART		0	II	none	3/3	f	72	Mid-frontal cortex
Control-3		PART	CAA widespread, mild	0	I	none	3/3	m	71	Mid-frontal cortex
Control-4	cognitive impairments	PART	CAA focal, mild to moderate	0	II	none	3/3	m	75	Mid-frontal cortex
Control-5		PART		0	I	none	2/3	f	73	Mid-frontal cortex
Control-6	metastatic cancer		rare Aβ plaques in neocortex	1	0	none	3/3	m	46	Hippocampus, Striatum
Control-7	Unverricht Lundborg disease			0	0	none	2/3	m	65	Hippocampus, Striatum
Control-8	metastatic melanoma		AD low	2	0	none	3/3	m	61	Hippocampus, Striatum

APOE apolipoprotein E, ARTAG aging-related tau astrogliopathy, AD Alzheimer’s disease, CAA cerebral amyloid angiopathy, CBD corticobasal degeneration, FTD frontotemporal dementia, FTLD frontotemporal lobar degeneration, LBD Lewy body disease, LATE limbic predominant age-related TDP-43 encephalopathy, PART primary age-related tauopathy, PiD Pick’s disease, PPA primary progressive aphasia, PSP progressive supranuclear palsy.

## Data Availability

The data presented in this study are available upon reasonable request from the corresponding author.

## Supplementary Materials

The following supporting information can be downloaded at https://www.mdpi.com/article/10.3390/ijms241813676/s1.
