# Supplementary material for: Novel Conformation-Dependent Tau Antibodies Are Modulated by Adjacent Phosphorylation Sites
Source: Int J Mol Sci. 2023 Sep 5;24(18):13676. doi: 10.3390/ijms241813676 (PMC10530490; doi:10.3390/ijms241813676)
Supplement: Supplementary file 1 [file ijms-24-13676-s001.zip › ijms-2539524-supplementary.pdf]

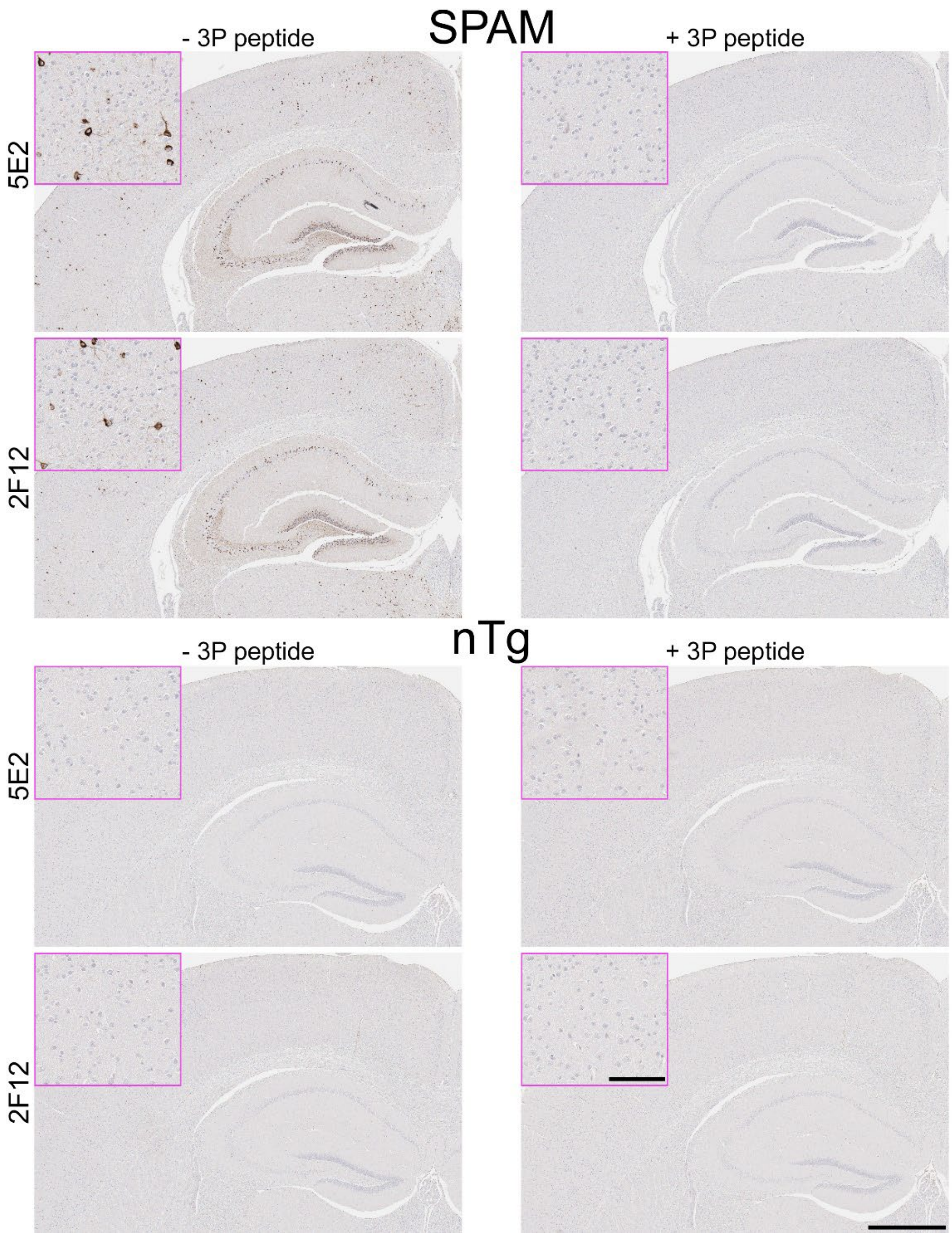

Supplementary Figure S1. Blocking of the 5E2 and 2F12 epitopes by 3P peptide pre-absorbed demonstrated by immunohistochemistry of SPAM and nTg mice. Brain tissue from SPAM and nTg mice was immunostained with 5E2 and 2F12 that was not incubated with 3P peptide (-3P peptide) or incubated with the 3P peptide (+3P peptide). High magnification images are of cortex from SPAM and nTg mice. Scale bar for high magnification images is 100µm. Scale bar for low magnification images is 700µm.

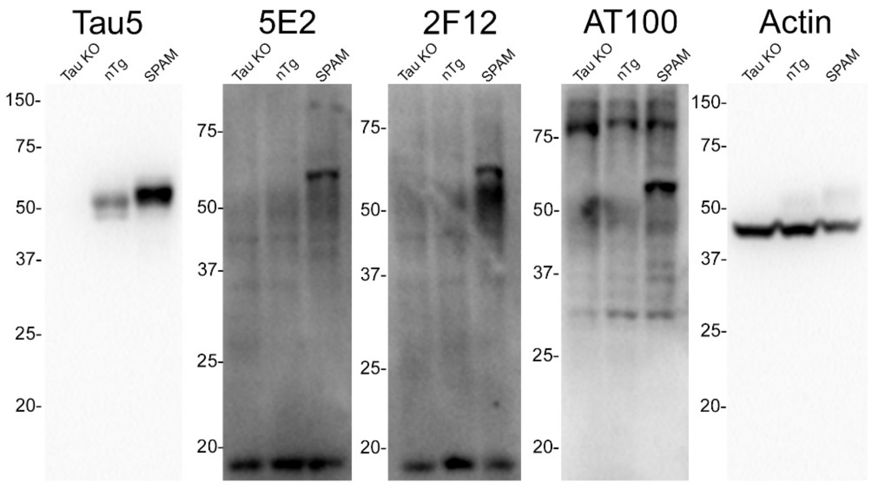

Supplementary Figure S2. 5E2 and 2F12 immunolabelling under denaturing conditions. Immunolabelling of tau protein from brain lysates was performed using novel antibodies 5E2 and 2F12, as well as Tau5, AT100, and actin as a loading control. Relative mobilities of molecular weight markers are indicated on the left side of each blot.

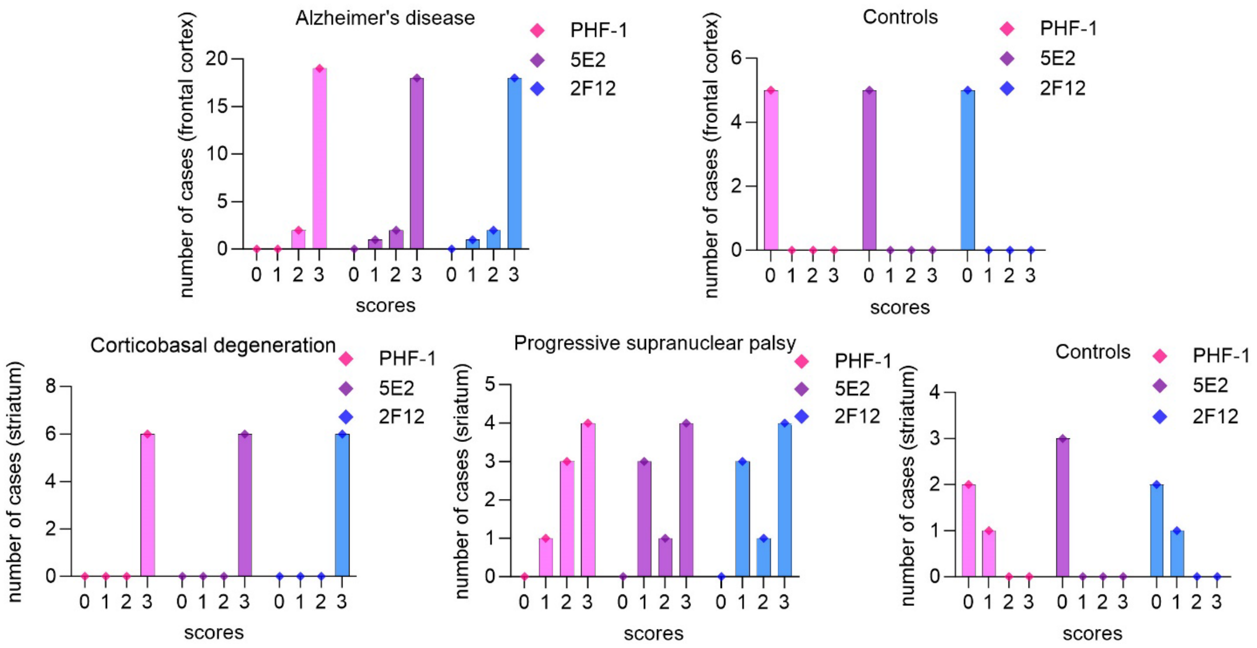

Supplementary Figure S3. Semi-quantitative analysis of human brain tissue staining with PHF-1, 5E2, and 2F12.

Frontal cortex tissue from AD (N=21) and control patients (N=5) as well as striatum from CBD (N=6), PSP (N=8), and controls (N=3) were independently scored by two investigators for overall tau neuropathology load.
